# Surgery

**Published:** 1859-09

**Authors:** Samuel W. Gross


### Bi-Monthly Abstracts;

OR, BI-MONTHLY NOVELTIES IN MEDICAL, PATHOLOGICAL, SURGICAL, OBSTETRICAL, PHYSIOLOGICAL, AND CHEMICAL SCIENCE.

### SURGERY.

BY SAMUEL W. GROSS, M.D.

I.—*History of the Compressed Sponge as a Remedial Agent for the Treatment of Strictures of the Rectum, Urethra, etc.; also, of Morbid Growths and other Affections.* By J. P. Batchelder, M.D. (New York Journal of Medicine, May, 1859.)

The author of this paper has, of late years, devoted much attention to the treatment of surgical affections by the compressed sponge, but seems to place more confidence in the article than it probably deserves. The most remarkable portion of his paper is that pertaining to the treatment of malignant tumors: one case of scirrhous breast, two of cancer of the lower jaw, and one case of cancerous inguinal glands being perfectly cured, while several other cases were much improved. For the details of these cases we must refer to his article, of which the following is a brief abstract:—

In preparing the plates of sponge, Dr. Batchelder uses a fine and dry article, and compresses it by means of a common copying letter-press, or a heavy weight, under which it is to be kept until wanted for use. The other form is that of a tent, which is made by transfixing a fine wet sponge with an awl, and wrapping it firmly with a cord or packthread; when it dries, the awl is removed, and the tent is ready for use; or the sponge is moistened with mucilage, and prepared in the same manner, being, when dry, trimmed smooth with the knife. The latter is applicable to cases in which the tent is difficult of introduction, and the moisture of the parts might produce its premature expansion, as in dilatation on the canal of the neck of the uterus. When employed in internal parts, the tent should be attached to a cord to facilitate its removal.

The author first applied compressed sponge, with marked success, to enlargement and abscesses of the female breast, and reports of cases, published in late years, fully confirm its utility. The object of the present paper is to point out the various surgical affections in which it may be employed.

1. *Dilatation of the Canal of the Cervix Uteri for the relief of Sterility, difficult Menstruation, and other Affections.*—The tent, prepared with mucilage, is carried up to the os with a pair of long-bladed forceps, or a stilet, constructed expressly for the purpose, and, by slight pressure upon its base, is made to enter the cervical canal. It should be removed every twenty-four hours to prevent irritating and offensive secretions, and the vagina should be well syringed with water. The speculum will sometimes be useful; and as the canal of the cervix is occasionally very small, it will, as a preliminary measure, be necessary to enlarge it with fine metallic dilators, until a small tent can be introduced. When the object is to induce premature labor, the tents should be worn continuously, and be gradually increased in size. In pregnancy, complicated with hemorrhage, when the os is partly open, but unyielding, and the labor is tardy, the tent, by making a good tampon, stops the flow of blood, excites the dilatation of the os, and also uterine contractions.

2. *Dilatation of Sinuses.*—In most cases of sinuses the sponge-tent, by producing a change in their lining membrane and a free purulent discharge, effects a cure. From time to time the tent should be made larger.

3. *Anal Fistule.*—In abscess of the ano-rectal region, a free and early incision should be made, and a sponge-tent introduced to favor the free discharge of pus and the healing of the cavity from the bottom. When the abscess has opened spontaneously, and formed an incomplete fistule, a tent introduced into the cavity fulfills the same indications, and by keeping the external orifice open, prevents the collection of pus, which, by its pressure, would produce the absorption of the septum between the rectum and cavity of the abscess, and thus producing an internal orifice, a complete fistule would be formed. When, therefore, there is no internal opening, the method will be found successful.

4. *Diseased Bone with Fistulous Passage.*—In most of the cases of caries or necrosis of the bones in which the author has tried the sponge-tent, a cure has resulted without a resort to the knife or scraping instruments. In a few weeks the sinus can be dilated to such an extent as to admit of the introduction of several fingers, or a pair of forceps, for the removal of the sequester or the ulcerated bone, by the repeated application of the sponge. Several cases of strumous or syphilitic enlargement of the bones have been cured by plates of compressed sponge, aided by appropriate constitutional remedies.

5–6. *Dilatation of the Meatus Auditorius, and Affections of the Nasal Cavities and Bones.*—In cases of contraction of the external auditory canal, the sponge-tent may be used with advantage, as well as in ulceration, contraction, and other affections of the nasal bones and cavities.

7. *Dilatation of Strictures of the Rectum.*—The author mentions three cases of rectal stricture treated with the sponge-tent, one proving fatal, as the disease was malignant, the other two being cured. The tent should be prepared with mucilage, if there be much irritability, being used larger as occasion requires. When the irritability and sensitiveness of the part are great, it will be well to precede the use of the tents by anodyne suppositories.

8. *Internal Piles.*—The tent, introduced just within the rectum, may prove useful in bleeding piles, or hemorrhage of this portion of the intestine.

9–10. *Stricture of the Urethra and Dilatation of the Female Urethra.*—In cases of urethral stricture a metallic sound should first be pressed against the resisting part; when being withdrawn, a tube, containing a transfixed tent and a piston, is carried down, and by pressure on the piston, the tent is made to enter the stricture When it is firmly in place, a few drops of water, or if there be irritability, of an anodyne solution, are passed down the tube to moisten the sponge. The cord attached to the tent should be guarded with a button to prevent its passing back into the bladder. It is best to allow the expulsory power of the bladder, in micturition, to remove the tent; should this not succeed, gentle traction may be made. When it is deemed necessary to dilate the female urethra for the removal of foreign bodies from the bladder, the sponge-tent will be found to answer every purpose.

11. *Morbid Growths.*—The compressed sponge, by its firm and equable pressure, induces the absorbents to remove tumors, whether benign or malignant, and should be confined in its place by the bandage, both being kept constantly wet. In case of malignant growths, if not removed entirely, they may be rendered harmless for years; and the author reports, in support of this opinion, a case of scirrhous tumor of the breast, completely cured in about two months; a case of scirrhous growth of the breast and a scirrhous nodule on the outer edge of the pectoralis major, in which the nodule entirely disappeared, and the tumor of the breast had greatly diminished, when the patient was lost sight of; a case of ulcerated malignant tumor of the breast, with well-marked cancerous cachexia, in which a sponge-tent was kept in the fistule, dilating it to a large size, with the result of a diminution of the tumor and a great improvement in the general health. The patient was lost sight of, but probably died. In three other cases of malignant disease of the breast, it produced diminution of the tumors, and prolonged life. In two cases of malignant disease of the lower jaw, (epulis?) both patients were cured. It has also been found useful in the removal of the callous edges of ulcers.

12. *Cancer of the Penis, with enlarged Glands of the Groins.*—The penis was amputated, and the glands removed with the compressed sponge; the patient's health was much improved, and the cancerous cachexia disappeared.

13–14. *Swelled Testicle and Vegetations.*—The testicle or penis is to be turned up over the pubes, and retained in position by the spica bandage, which, with the sponge, is kept constantly wet. The author has great confidence in emesis in the case of swelled testicle, and in that of warty excrescences he draws off the urine with a flexible catheter, so as not to disturb the dressings.

15. *Non-malignant Tumors.*—Enlarged lymphatic glands, especially buboes, are speedily removed by this means. Dr. Batchelder states that, in the last fifteen years, he has had but two cases of suppurative bubo, and these were owing to the imprudence of the patients. In addition to the sponge and bandage, in these cases, he recommends a hernial truss with a glass pad and strong spring, the pressure being properly graduated.

16. *Enlarged Joints.*—In cases of effusion within the joints from synovitis, or in effusion from any cause outside the joints, the sponge has succeeded, especially in the latter, better (in the author's hands) than any other means.

17. *Compressed Sponge as a Styptic.*—In wounds of certain arteries it is not always practicable to apply a ligature, and the surgeon relies, for a time at least, on compression In regard to wounds of the palmar and plantar arteries, the author says: "The phenomena attending these wounds are often somewhat peculiar. The blood forced extensively into the surrounding cellular substance, perhaps under the aponeurosis, comes welling out from a considerable surface, which renders it quite difficult to find the wounded vessel. If this surface be scraped again and again with the handle of a scalpel, the extent from which the blood issues will be lessened down to a small space." Upon this he places a small piece of pointed compressed sponge, confining it by means of a compress and a roller, and flexes the arm at an acute angle, a position which renders pulsation at the wrist almost imperceptible.

18. *Pachydermatocele.*—This affection, first described by Dr. Mott, is congenital, and consists of a hypertrophous condition of the skin and subcutaneous cellular tissue. Dr. Batchelder has seen three cases of this disease, which were operated upon by Dr. Mott, and in all the affection returned. After its recurrence in one of these cases, in which the mass hung in folds on the side of the face, the author applied, not without some difficulty, the compressed sponge and roller. In about two months, the whole mass, excepting a small portion near the eye, which had resisted the treatment more than any other part, had become absorbed, and the patient, thinking the rest would disappear, ceased to consult the author. In a few months, however, he returned, the mass being larger than before. A renewed and persevering application of the same means effected a cure.

19. *Varicose Veins.*—In one case the author applied the compressed sponge over the internal saphenous vein, and confined it by a vulcanized India-rubber bandage. The dressings were worn only through the day, and had the effect of relieving the symptoms, and rendering the patient's locomotion much more comfortable. It is not stated whether a cure was effected.

II.—*Some of the Causes which have occasionally rendered Excision of the Knee-joint more or less unsuccessful.* By P. C. PRICE, Esq. (Med. Times and Gazette, Nos. 459, 460, April, 1859.)

This interesting surgical contribution will be found to contain all the cases of excision of the knee-joint that have occurred in Great Britain during the past eight years, that is, during a period extending from July, 1850, to the end of 1858. They amount to 160, and from these the author shows some of the causes of failure from the operation, and other valuable facts, thus lending his assistance in giving this operation the position to which its importance undoubtedly entitles it.

Of these cases, the mortality was in the ratio of one death to five recoveries, thirty-two cases proving fatal; the result being the same as that obtained by Mr. Butcher in one-half the number of cases. The age of the youngest patient was three years, and that of the oldest forty-seven years. Of the entire number but six or seven were operated on for deformity, and one only for accident, leaving 152 cases in which the procedure was instituted for extensive destruction of all the structures of the joint. In some of these it was practiced as a final resort, and in some amputation should have been performed. Taking, however, all the cases into consideration, the different surgeons had evinced nice judgment in the selection of their subjects. When the operation was completely unsuccessful, it terminated either in death or in amputation of the limb; when partially so, the member remained more or less useless. In eighteen cases amputation was required at a period more or less remote from the primary operation, and in one instance only was the result fatal.

The causes of death in the 32 fatal cases were in 8 pyæmia, in 6 exhaustion, in 5 irritation, in 4 shock, in 1 dysentery, in 1 suppression of urine, in 1 pleuro-pneumonia, in 1 erysipelas, in 1 peritonitis, in 1 acute phthisis, in 1 after-amputation, and in 2 the causes were unknown. The age of the youngest patient who died from one or the other of these causes was five years, that of the oldest thirty years.

From the above it will be seen that pyæmia was the most frequent cause of death, proving fatal in one-fourth of the cases. Mr. Bryant has lately pointed out that pyæmia was the principal source of fatality in 300 amputations of the upper and lower extremities, in 43 per cent. of all fatal cases, and in 5·4 per cent. of all pathological amputations.

In comparing the results of amputation for disease with those of excision of the knee-joint, it will be perceived that the percentage of recoveries is in favor of the latter. Thus, Mr. Teale has shown that in 160 amputations of the thigh, for disease, in the London hospitals, during a period of three years, 38 proved fatal, or 1 in 4½; and of 134 amputations of the thigh, for disease, in the provincial hospitals, during the same period, 33 resulted in death, or about 1 in every 4 cases. As before stated, the mortality of excision of the knee joint is in the proportion of 1 death to 5 recoveries, which corresponds to the statistics of Guy's Hospital, as drawn up by Mr. Bryant, who states that "1 case in 5·5 was about the proportion of fatal terminations in pathological amputations. For chronic disease of the articulation, amputation was more favorable, the mortality standing at about 1 in every 7 cases."

Of the wholly unsuccessful cases which were not attended with death, it was found necessary to amputate the thigh in eighteen instances, with the remarkable result of only one death. The causes requiring this measure were,—

1. "The occurrence of non, or insufficient union, associated with abscesses of the soft parts, with necrosis of the ends of the bones." This condition is owing to the want of a proper selection of cases; thus it is well known that those cases of tubercular infiltration of the extremities of the bones, leading to the destruction of the joint, are never suitable to an operation, as osseous, or even fibrous union, will seldom take place, and the limb will be the seat of persistent sinuses, which, by exhausting the patient's system, will imperatively call for the removal of the limb.

2. "The existence of abscesses, more or less acute, accompanied with hectic, night-sweats, diarrhœa, etc." For the local complications and the constitutional disturbance, amputation was required in eleven cases, the earliest operation being nine days after the excision, the latest two years and three months.

3. "Erysipelas and other specific affections, as measles, etc." General diseases and erysipelas required amputation in four cases, all of which made good recoveries.

4. "Inappropriate management of the limb during treatment." In three instances amputation was performed on account of bad management of the limb and the impossibility of keeping the extremities of the bones in apposition. It is a matter of the utmost importance to maintain the limb in a proper position during the after-treatment, and this being well attended to the frequency of amputation will be diminished.

Partial success has often followed the excision of the knee-joint, the limb being more or less useless as regards locomotion, although the deformity or disease may have been removed. The causes of the partial success are,—

1. "Imperfect union of the divided ends of the tibia and femur arising from a peculiar diathesis pervading the constitution." Mr. Price states it as his opinion that when the cancellated structure of the bones is much involved in the disease, the bond of union will at first be fibrous and flexible, which, at a later period, may become bony in its nature. It is not necessary, as is generally supposed, that bony anchylosis should take place for a good use of the limb, although such an occurrence is not infrequent.

2. "From the non-maintenance at rest, or inappropriate treatment of the part immediately concerned in the operation." As before stated, the perfect quietude of the part, and a suitable mechanical after-treatment, are indispensable to a proper uniting medium. Without attention to these points nothing can be expected from the operation.

3. "From taking away too great an amount of bony structure." The surgeon should be very cautious, especially in the case of children, not to remove the whole of the epiphyses of the bones, as by so doing arrest of development will be sure to follow, and as the patient advances in years, the limb will become shorter than the sound one. Attention to this point must, therefore, be insisted upon.

4. "From the occurrence or persistence of abscesses in the soft tissues only, or complicated with disease of the bone." This complication is a common occurrence in children, especially when there is a scrofulous taint of the system. Dead bone, or the remains of diseased synovial membrane, will favor the occurrence of sinuses and abscesses, which will, however, yield to a judicious and persevering treatment.

Swelling of the ankle and leg is rather a common occurrence, but need not give rise to any fear as to the result of the operation, as it will generally disappear by appropriate management.

III.—*On Two Modifications of the Operation for Cataract.* By Dr VON GRAFE. (Communication made to the "Verein der Berliner Aerzt." Allgemeine Medizinische Centralzeitung, 20, 1859.)

One of the most common operations for cataract, that of extraction and the formation of a large flap, affords about ten favorable results in twelve cases; in one case only a partial restoration of sight is procured, and in the other suppuration of the eye is caused by loss of the flap. Division of the lens, which has been proposed as a substitute to the above method, is only applicable in cases where the lens is capable of being absorbed; when the nucleus of the lens is hard and yellow, it is apt to be followed by iritis, and must, under such circumstances, give way to extraction. In the operation of reclination the lens acts as an irritant, and produces internal ophthalmia, increase of intra-ocular pressure, excavation of the optic nerve, and amaurosis. The statistics of the operation show that vision is preserved in only one-half of the cases. In old persons, therefore, extraction with the formation of a flap should be preferred to all other operations, which may, however, be used in exceptional cases.

The dangers of extraction are owing to the extensive flap of the cornea, on account of which comparatively little tissue is left for the supply of nourishment, which evil is further increased, and the mortification of the flap is favored by the contusion produced by the lens on its escape. This latter difficulty is obviated by the linear section; but this is only applicable in cases of soft cataract, as in hard ones the frequent introduction of Daniels' scoop causes dangerous irritation of the iris.

In order to render the linear section applicable to a greater number of cases, Dr. Von Gräfe has made the following modification: The linear incision is made in the sclerotica in such a manner as that the inner cut occupies the border between the sclerotic and cornea; the *iris is then drawn out and a portion of it snipped off, so as to form a broad coloboma.* A broad Daniels' scoop is pushed behind the lens, which, being broken up into small pieces, is removed, without fear of subsequent irritation of the iris. This operation is suitable to soft cataracts with a moderately large and hard nucleus, and is to be preferred in old persons, in whom the state of the constitution would give reason to apprehend mortification of the cornea in the flap operation of extraction. The coloboma, which has but little influence on the sight, should be made on the temporal side instead of above, where it would be less visible, but would, in this situation, render the different steps of the operation more difficult.

The second modification proposed by Von Gräfe has reference to the operation of division of the lens. The operation consists in making an upward coloboma, eight days before the division of the lens. This preparatory measure has, as Von Gräfe alleges, the advantages of being followed by less swelling of the lens, one of the dangers of the other operation; larger wounds can be made in the capsule, and absorption takes place more readily, being effected in from four to six weeks, while in the simple operation of division it takes as many months.

IV.—*A New Mode of Dressing in the Wounds of Amputation.* By M. Laugier. (Journal de Médecine de Bordeaux, June, 1859, and Comptes Rendus de l'Académie des Sciences.)

The objections made by the author to the ordinary manner of dressing stumps with the roller and adhesive strips, are that the bandage does not fulfill the indication of causing the flaps to unite from the bottom, that its compression, which must be moderate for fear of producing strangulation of the stump, yields to the weight of the limb, placed on the pillow or bed in such a position as tends to separate the lips of the wound, and that on account of its becoming loose it has often to be reapplied. The inconveniences of the adhesive strips are that they push back the fleshy portion of the flaps, and thus tend to favor the protrusion of the bone and the formation of a depot of pus; they sometimes cause erysipelas, and do not give an equable support to the part, which should be made to heal from the bottom toward the edge of the wound, or from behind forward.

With a view to maintain all portions of the flaps in accurate apposition, and to bring about immediate union, M. Laugier proposes to place under the bandage two plates of cork, of two lines in thickness, and of a length and width sufficient to permit it to embrace nearly the whole stump, from its base to its apex, and allow it to extend about two and a half to three inches over the free extremity of the stump. Its edge is then digitated and pierced with a number of openings to receive a piece of tape, which brings the opposed surfaces together at the end of the dressing. Previous to applying the cork, the stump is encircled by thick, circular pieces of amadou or tinder, which serve the purpose of rendering the pressure more easy and efficacious, and keeping the digitations of the cork from the parts. A pledget of lint, spread with cerate, is then placed on the wound, and the opposite portions of the cork are approximated by tying the pieces of tape. No sutures are used. The stump is thus enveloped in a firm case, which keeps off pressure from the wound, and permits the patient more easy and free motion. At each dressing it is not necessary to take it off; the tapes are untied and cleanliness is effected.

The advantages claimed by M. Laugier are that the dressing causes union from the bottom of wounds made in amputation of limbs in their continuity, it sustains the fleshy portions against the bone, insures the direction given to the lips of the wound, does away with the inconveniences of adhesive plaster, and, finally, it protects the stump from exterior shocks, and gives greater latitude to the movements of the patients and the amputated limb.

V.—*I. Report of Five Cases of Fracture of the Vertebræ.* By CHARLES PHELPS, M.D., House Surgeon to Bellevue Hospital. (New York Journal of Medicine, January and March, 1859.)

*II. A Series of Cases of Injury of the Spine.* By F. BIRKETT, Esq. (British Medical Journal, March 26th, 1859.)

In these ten cases we find many points of interest in regard to the symptomatology and treatment of fractures and dislocations of the vertebræ, and the effects produced by such injuries on the spinal cord. Two of these cases only eventuated in recovery, thus showing how little can be expected from treatment, however judiciously selected. In the second case, the depressed portion of bone was removed, an operation which was first performed by Mr. Cline, of London, and which has also been resorted to in this country by Drs. Smith, J. Rhea Barton, and Goldsmith. Altogether, it has been done about a dozen times with no marked benefit, and yet it should always be tried when deemed advisable.

I.—CASE 1. *Fracture and Luxation of the Seventh Cervical Vertebra; death at the expiration of a hundred hours.* (Service of DR. WOOD.) An Irishman, thirty years of age, of intemperate habits, but good constitution, fell down a flight of stairs, striking upon his back, and was rendered insensible for a few moments. Upon the return of consciousness, he felt some numbness and incapacity for motion, but labored under no symptoms of injury of the spine or head. On admission, twenty-four hours afterwards, an examination showed complete paralysis and loss of sensibility of the entire body below the third rib. Sensation was perfect in both arms, but power of motion was lost in the left. The patient rested on his back, with the head and neck thrown forward, the latter of which was tender on pressure posteriorly. The respiration was abdominal, the temperature normal, the pulse but little affected, the penis was erect, and the fæces and urine had not been passed since the accident. On the following day a bedsore began to form on the nates, the temperature of the body was increased, the respiration was embarrassed and ten to the minute, and the speech was quick and abrupt. The bedsore was attended to, and a grain of morphia was administered every two hours during the day. On the following day the condition was much the same; the tympanites had increased, there was some hematuria, and he could retain but little nourishment. The morphia was continued, and an enema ordered. During the evening there was great dyspnœa; the injection had acted, and the hematuria ceased. On the next day there was dullness at the lower portion of the right lung, but no difficulty in breathing, and he was at times delirious. At seven P.M. both arms were devoid of sensibility, and paralyzed, the urine was high colored, and the tympanites increased. At nine o'clock he was unconscious; the body was hot, pulse full and strong, eyes suffused, and the lower jaw moved convulsively. In three hours he quietly died; and, on a post-mortem examination, there was found a complete transverse fracture of the seventh cervical vertebra and of its right transverse and articulating processes, with rupture of the ligamenta subflava. The bone was luxated backward, but the cord was not lacerated. The brain was slightly congested, and the medulla oblongata in a greater degree. The other organs were healthy.

CASE 2. *Fracture of the Body of the Tenth Dorsal Vertebra; operation for the elevation of the depressed portion; large effusion of blood within the theca; death.* (Under the charge of DR. STEPHEN SMITH.) An Irishman, forty-one years of age, of temperate habits and good constitution, on the twelfth of October was thrown from his cart, striking upon his back, and did not feel hurt until he was moved, when he suffered great pain, and found that he was paralyzed. He was admitted into the hospital two hours after the injury, in a collapsed state, the pulse being exceeding frequent and feeble, and the respiration eighteen a minute. The whole body was paralyzed and devoid of sensibility below the sixth intercostal space; there was severe pain in the back of the neck, although no bruise could be discovered, and numbness and tingling pain in the arms. The penis was slightly erect, the temperature of the body was natural, and a space of about an inch and a half in width was discovered between two of the spinous processes of the dorsal vertebræ. Stimulants and anodynes were freely used, and the urine was drawn off with a catheter.

On the following morning the pulse was 112, the respiration 26, and mainly abdominal, the temperature of the body was exalted, and the pain and numbness in the back and arms increased. In the afternoon sloughs had commenced on the feet, and, upon a consultation, it was determined to trephine the vertebræ. The patient being under the influence of chloroform, Dr. Smith made an incision six inches in length over the dorsal vertebræ, and found the subcutaneous cellular tissue filled with extravasated blood, and the lamina of the right side of the tenth dorsal vertebra depressed. This was removed with the trephine, and was followed by an escape of from six to twelve ounces of dark blood. The case soon terminated fatally, and an autopsy revealed "a fracture of the body of the tenth dorsal vertebra upon the right side, extending from the base of the transverse process half way to the mesian line anteriorly, without displacement—fracture of the arch of the vertebra upon the right side, with depression, and extravasation of blood to a large amount, extending from the lower cervical vertebra to the sacrum. Considering the increasing paralysis, the extravasation was probably still extending upward at the time of the death of the subject."

CASE 3. *Fracture and Luxation of the First Lumbar Vertebra; union of fracture; death after thirty-seven days.* This case, under the care of DR. STEPHEN SMITH, occurred in a healthy Irish woman, thirty years of age, who, on the evening before her admission, while asleep, fell out of a third-floor window, striking her left side upon a slate-roof two stories below. She was not rendered insensible, nor had she, previous to her admission, any symptoms of spinal lesion, excepting intense pain upon motion, and numbness in her left leg. An examination showed a backward displacement of one of the lower dorsal or upper lumbar vertebræ, with slight tenderness lower down. The respiration was natural, and the pulse a little frequent. For a few days the urine was drawn off, but soon passed involuntarily. A bedsore formed on the sacrum, which finally exposed the bone; the pain on motion gradually diminished, and at no time was there paralysis or insensibility of the body or extremities. Diarrhœa, however, supervened, and the patient died from exhaustion, thirty-seven days after admission.

Upon a post-mortem examination, the body of the first lumbar vertebra was found crushed, and the bone was completely dislocated backward. The fractured bone had thoroughly united.

CASE 4. *Luxation and Fracture of the Seventh Cervical Vertebra; resulting in death.* (Under the care of DR. WOOD.) A robust, but intemperate Irishman, forty-two years old, was admitted into the hospital on the sixteenth of July, having the evening before been thrown from his cart upon his head and back, with the effect of immediately paralyzing his limbs and causing severe pain, especially on motion, in the lower cervical region. He had cramps in his left leg, and his bowels and bladder were evacuated voluntarily. The paralysis was complete below the clavicles; the intellect was clear; there was some dyspnœa; the respiration was 20, and mainly abdominal; and there was no pain in the back of the neck, except on motion. Beef tea and port wine were ordered, and during the evening the patient was comatose. On the following day, at ten in the morning, he was still comatose, and at three P.M. the face became turgid, the eyes protruded, the lips were purple, and three quarts of blood flowed from the mouth. He died without any convulsions. The coroner refused permission to hold an autopsy.

CASE 5. *Fracture and Luxation of the Fifth Cervical Vertebra; recovery.* (Service of DR. STEPHEN SMITH.) An Irishman, twenty-nine years of age, and of good constitution, in September, 1856, while intoxicated, fell down a flight of stairs, striking the back of his head and neck, being rendered unconscious and remaining so for three hours. He had no paralysis, anæsthesia, or other symptom of lesion of the spine, but experienced pain in the neck on motion, and suffered from cephalalgia.

In August, 1857, he was again admitted on account of the pain in the head, which had never left him. Upon examination externally, Dr. Smith found the fifth cervical vertebra luxated forward, and the bone could be felt forming a large prominence in the pharynx. The neck was firm in its new position, which was awkward, and he had some difficulty in swallowing solid food.

II. — Case 1. *Dislocation and Fracture of the Third Cervical Vertebra; death.*—A strong, muscular man, thirty-two years of age, fell down stairs, while intoxicated, and was placed in bed in a perfectly helpless condition. He was admitted into Guy's Hospital on the following evening in a collapsed state, with great difficulty of breathing. There was complete paralysis and anæsthesia of the body below the clavicles, and his bladder was distended. He reacted slightly, but died at the end of thirty-six hours.

On an autopsy the neck was found to be contused, the third cervical vertebra was luxated, and the anterior common ligament ruptured. The laminæ of the second and third vertebræ were broken and movable, and the right lamina of the atlas was fractured at its root. The cord was crushed and nearly torn across at the site of the dislocation; the lungs were congested, and the bronchia filled with bloody mucus.

Case 2 *Dislocation of the Fourth Cervical Vertebra; death.*—A male, seventeen years of age, during a fight of two hours duration, was thrown to the ground, and found some hours afterwards by a policeman. When admitted he was sensible, and the whole body below the neck was paralyzed; the breathing was abdominal and much embarrassed, and there was some sensation over the shoulder and upper part of the chest. He gradually sank, and died thirteen hours after the accident. On a post-mortem examination the muscles of the neck were found contused and ecchymosed; the fourth and fifth cervical vertebræ were separated from each other, and the ligaments ruptured. There was no fracture, and the membranes of the cord were uninjured, but the marrow was pulpified and filled with blood. The lungs were much engorged, and the bronchial tubes contained blood.

Case 3. *Fracture and Dislocation of the Eleventh and Twelfth Dorsal Vertebræ; death in four months and a half.*—A stout Irishman, thirty-one years of age, fell down the hold of a ship, striking upon his back, which caused paralysis and anæsthesia of the lower extremities. The two lower dorsal vertebræ were found to have been injured, and the fascia was stripped off several of the dorsal spines. He was suffering intense pain; and while under the influence of chloroform, extension was applied, but without effect. On the third day he had some feeling in his legs, but no motion; suffered great agony on the slightest movement, and was withal cheerful, and had a good appetite. The urine was drawn off twice a day, and croton oil was administered to relieve the costiveness. In a month, bedsores had formed, and he gradually sank, and died four months and a half after the reception of the injury.

An autopsy revealed complete separation of the eleventh and twelfth dorsal vertebræ, with fracture of their articulating processes. The cord was disintegrated, softened, and of a brownish color at the point of injury; the bedsores over the sacrum had exposed the spinal canal, and the kidneys were filled with pus.

Case 4. *Fracture and Dislocation of the Twelfth Dorsal Vetebra; death on*

*the eleventh day.*—A laborer, twenty years old, fell through a trap-door, and in attempting to walk, felt great pain in the loins, and had no use of his legs. The constitutional disturbance was considerable, and the bladder was paralyzed. On the night of admission he vomited several times, and the bowels had not acted. During the next four days, high constitutional symptoms supervened; he passed bloody urine, and gradually sank until the eleventh day, when he died. Stimulants, nourishment, and opium, formed the treatment.

On a post-mortem examination, the eleventh dorsal vertebra was found to be slightly luxated, its body crushed and broken in half, and its arches fractured and quite movable. The cord was softened, crushed, and inflamed opposite the injury, and the bladder was ulcerated and covered with phosphates.

CASE 5. *Fracture of the Spine about the Eleventh Dorsal Vertebra; recovery.*—A healthy laborer received a blow upon his loins from a heavy sack falling from a height of about nine feet, while he was in a stooping position. He was immediately rendered powerless, and had no feeling in his legs. On admission, he was sensible; both legs were paralyzed, the left having no sensation, and the right being excessively sensitive. The urine had to be drawn off with a catheter, and on the third day the feeling in both legs was almost natural. In three weeks he passed his urine voluntarily; and in six weeks he was able to move his toes slightly. At this time, upon examining the back, the spine of the eleventh dorsal vertebra was found much displaced. He continued to improve slowly, and left the hospital in about three months and a half from the time of admission. The treatment consisted in tonics and opium.

VI.—*Cure of a Popliteal Aneurism by forced Flexion of the Knee.* By M. MAUNOIR. (Echo Médicale Suisse, Sept., 1858; Archives Générales de Médecine, July, 1859.)

In our last number we presented to our readers a brief abstract of two papers on the cure of popliteal aneurism by flexion of the knee-joint, and we have now to chronicle another case under the charge of M. Maunoir. The aneurism was as large as the fist, and was seated in the lower portion of the popliteal space. The author perceiving that forced flexion of the leg stopped the pulsations of the tumor, advised the patient to keep the same position, and confined the limb by an appropriate bandage. In a few days the pulsations were less forcible, and in twenty days a cure was effected. But little inconvenience attended the treatment, and when the patient was seen at the end of a year, there existed a small, hard tumor, about the size of a pigeon's egg, in the popliteal region, the remains of the previous disease.

VII.—*On Emphysematous Tumors of the Temporal Region.* By DR. COSTES. (Journal de Médecine de Bordeaux, Oct., Nov., Dec., 1858.)

Professor Costes, of Bordeaux, has undertaken the task of collecting the few and scattered cases of this affection which are on record, and has made them the subject of a very interesting paper. The result of his investigations is contained in the following conclusions:—

1. Although of rare occurrence, emphysematous tumors, extending more or less to the neighboring parts, are observed in the temporal region.

2. They originate in consequence of an erosion or destruction of the exterior lamella of the mastoid process, and are formed by the air, which, in the normal state, is contained in the cavity of the tympanum, and in the cells of the mastoid process, escaping from underneath the pericranium, and filling the meshes of the surrounding cellular tissue. Murray conclusively demonstrated, in 1838, that all the cells of the mastoid process communicate with each other, and open into the cavity of the tympanum.

3. Palpation of such a tumor produces a characteristic crepitating sound. The change in the structure of the bone which gives rise to this tumor manifests itself generally in the form of a pointed projection, an osteophyte, etc.

4. These tumors are, in general, more or less reducible; the reduction takes place with a noise, which the patient distinctly perceives in the respective ear.

5. The cause of these tumors seems to be an excessive development of the mastoid cells, and an extraordinary tenuity of the external lamella of the bone which covers them. The real cause of the structural change in the bone which produces the tumors in question is still unknown.

6. These tumors are developed very slowly, and remain for a long time without giving rise to pain; when they have attained a considerable size they produce troublesome symptoms.

7. Such tumors are in themselves without danger; in consequence, however, of complications, neglect, or wrong treatment, they may become a serious disease.

8. The treatment consists in opening the tumor by a superficial incision, in order to remove the air contained in the cellular tissue, and in bringing about an adhesion between the soft parts and the bone, so as to prevent a recurrence of the infiltration of air. The latter object may be obtained by introducing a small seton, or by making strong compression on the external parts; injections of iodine may also possibly be of benefit.

